# Congenital solitary reticulohistiocytosis (Hashimoto - Pritzker)^☆^

**DOI:** 10.1016/j.abd.2022.01.005

**Published:** 2022-09-21

**Authors:** Luciana Prates Nogueira de Lima, Carolina Viza Amorim, Rachel Martins Marinho, Maria Letícia Cintra, Elemir Macedo de Souza

**Affiliations:** a Dermatologist - specialist in Hansenology by the Brazilian Society of Hansenology, Piracicaba, SP, Brazil; b Department of Pathology, Universidade Estadual de Campinas, Campinas, SP, Brazil; c Institute of Pathological Anatomy, Piracicaba, SP, Brazil; d Department of Dermatology, Universidade Estadual de Campinas, Campinas, SP, Brazil

**Keywords:** Histiocytoma, Immunohistochemistry, Pathology

## Abstract

Congenital and self-healing Hashimoto-Pritzker reticulohistiocytosis is the benign variant of the Langerhans cell histiocytosis (LCH) group. It is characterized by multiple skin lesions (congenital or appearing during the first days after birth), without systemic manifestations and spontaneous resolution in days to months. The authors report the case of a boy with a single congenital leg skin lesion, a rare disease variant. Through histopathology, a dense skin infiltration of S100 protein-, CD1a-, CD207-immunomarked cells was found. KI67 index was high (62%). A complete spontaneous resolution occurred 07 days after the biopsy (25 days after birth). Monolesional disease, distal limb lesion, absence of lesions in the mucous membrane or seborrheic area, and less than 25 percent of LCs with Birbeck granules were said to be possible clues for a favorable prognosis in LCs histiocytosis. But, as a precautionary measure, the child will be followed up until at least 2 years of age.

## Introduction

Langerhans cell histiocytosis (LCH) comprises four clinical variants: Hand-Schüller-Christian, eosinophilic granuloma, Letterer-Siwe, and Hashimoto-Pritzker.

In common, they present proliferation of Langerhans cells, with Birbeck granules under electron microscopy and immunohistochemical positivity for S100 protein, CD1a, and CD207 (langerin).^1^

Congenital and self-healing Hashimoto-Pritzker reticulohistiocytosis is the benign variant of the Langerhans cell histiocytosis group. Multiple skin lesions, congenital or appearing in the first days after birth, without systemic manifestations, and spontaneous resolution in days to months, characterize the disease.^1^ The authors describe a rarer single lesion presentation.

## Case report

A newborn otherwise healthy boy was noted to have a single skin lesion on his left leg. He was conceived by in vitro fertilization, from unrelated parents, all prenatal serological tests were normal, and the childbirth took place through cesarean section, without complications. At birth, BCG and Hepatitis B vaccines were administered.

**Figure 1:**
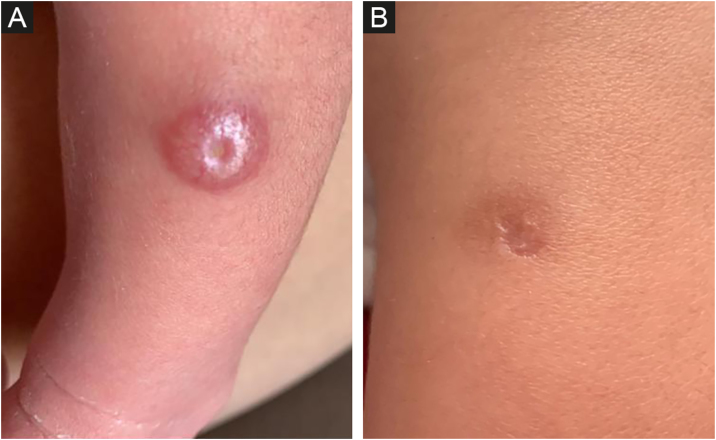
Hashimoto-Pritzker histiocytosis. (A) Congenital skin left leg papulo-nodular, erythematous-hyperchromic lesion, with an exulcerated center. (B) Complete self-regression 27 days after birth and 7 days after biopsy. Central scar area corresponds to the punch biopsy site.

The dermatological examination revealed, on the left leg (middle third, anterolateral face), a 1.2 cm papulo-nodular, erythematous-hyperchromic lesion, with an exulcerated center (Fig. 1A). After 10 days, there was no improvement with the use of antibiotics and the lesion was more infiltrated and shinier, measuring 1.5 cm in diameter, with central ulceration of 0.4 cm.

At 18 days of age, a biopsy was obtained and, after 7 days, the lesion showed a complete resolution, being replaced by a 1 cm hyperchromic macule, with a central scar area, corresponding to the punch biopsy site (Fig. 1B).

**Figure 2:**
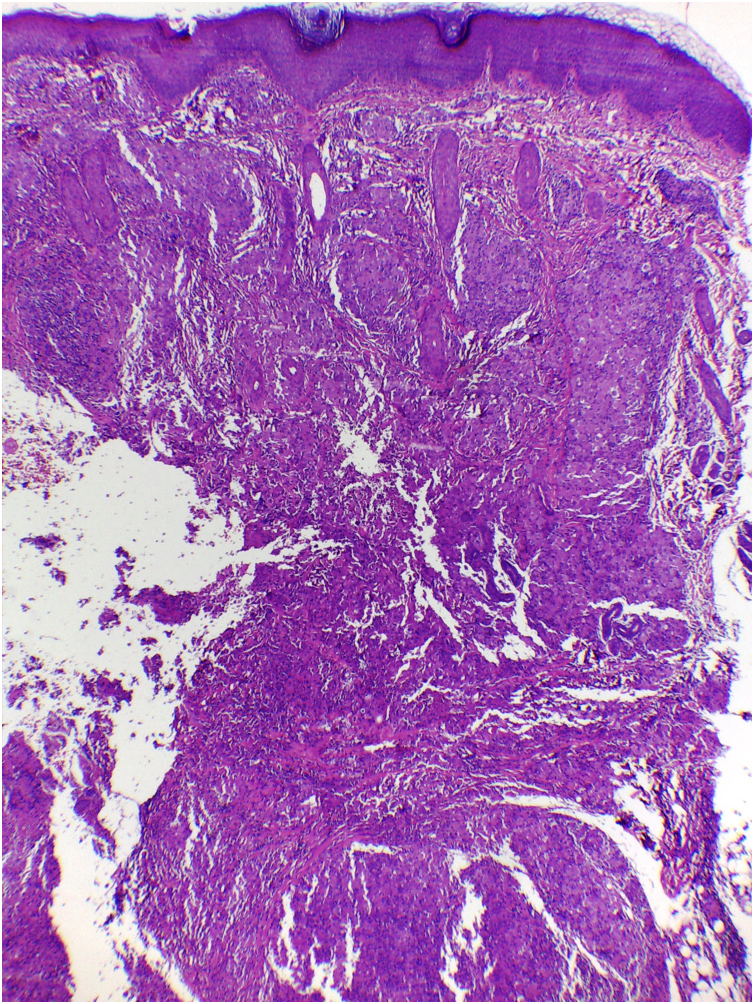
Hashimoto-Pritzker histiocytosis – histopathological view: dense dermal-hypodermal infiltration of histiocytoid cells (Hematoxylin & eosin, ×40).

**Figure 3:**
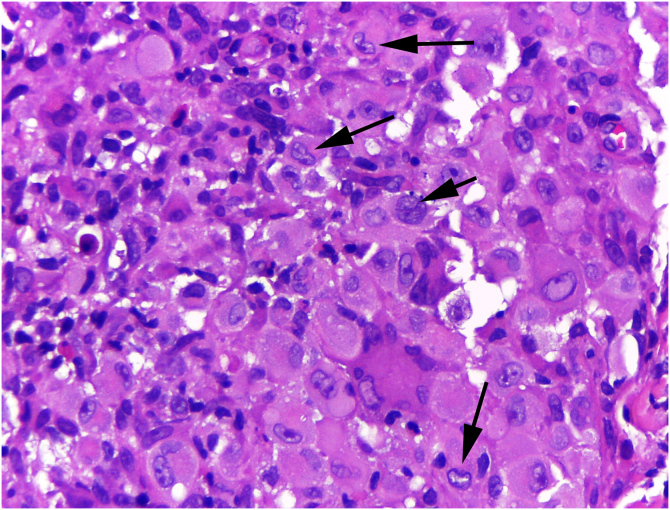
Hashimoto-Pritzker histiocytosis ‒ high magnification reveals cells with abundant eosinophilic cytoplasm and clear ovoid, or kidney shaped (arrows) nuclei (Hematoxylin & eosin, ×400).

**Figure 4:**
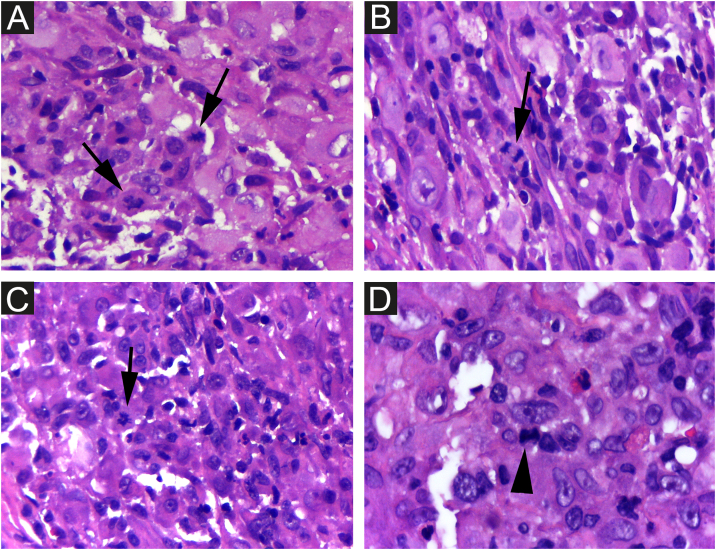
Hashimoto-Pritzker histiocytosis- There are several mitotic figures (arrows). Hematoxylin & eosin, ×400.

On histologic examination, the dermis and hypodermis were densely infiltrated by cells with abundant eosinophilic cytoplasm, clear ovoid or kidney-shaped nuclei, with several mitotic figures, admixed with a slight and variable number of eosinophils and lymphocytes (Figure 2, Figure 3, Figure 4).

**Figure 5:**
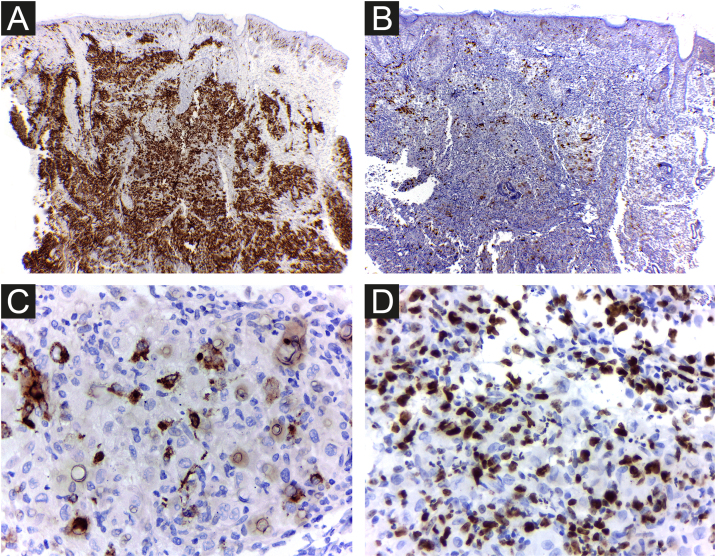
Hashimoto-Pritzker histiocytosis ‒ the cells stain strongly positive for CD1a (A) and less for CD207 (B and C). High proliferative Ki67 index (62%) (D) Original magnification ×40 (A, B); ×400 (C, D).

The cells were strongly positive for CD1a and S100 protein, and less for CD207; the Ki67 index was high (62%) (Figs. 5 A, B, C, D).

Routine laboratory tests, ultrasonography, and bone radiographs were normal, and no recurrence was noticed at 8 months of life.

## Discussion

Hashimoto-Pritzker’s reticulohistiocytosis (HPR) is characterized by multiple nodular dermatological lesions.^1^ However, the frequency of the solitary form is probably underestimated, perhaps due to the rapid spontaneous lesions’ regression, the non-specific clinical findings, and some of which go unnoticed and are not diagnosed. Zunino et al. reported 8 cases, and in the reviewed literature, they found 30% of cases with a single lesion.^2^

To set up the diagnosis of LCH is necessary the support of clinical, histological, and immunohistochemical data. The differential diagnoses considered in the presence of a congenital papulo-nodular lesion are congenital mastocytoma, eosinophilic granuloma, juvenile xanthogranuloma, congenital Spitz nevus, hemangioma, the cutaneous form of leukemia, or lymphoma, congenital fibrosarcoma, infantile hamartoma, and infantile myofibroma.^2^ A mixed infiltrate of inflammatory cells, containing macrophages with broad acidophilic cytoplasm and cleaved nuclei, is indicative of this condition. Immunostaining for CD1a and CD207 defines LCH. The same histological findings of the patient’s lesion may be seen in multifocal single-system or multisystem disease.^3^

On immunohistochemical stain of 7 HPR patients’ samples, it was found immunoreactivity for S100 protein and CD1a. The LCs phenotype was confirmed by the specific presence of Birbeck granules, on electron microscopy, performed in half of the cases.^2^ Langerin (CD207) is a surface receptor on Langerhans cells, a major molecular constituent of Birbeck granules. The CD207 immunomarking allows ruling out the possibility of indeterminate histiocytosis.^3^

It is difficult to predict the severity of the disease based on initial clinical and histological findings. There is an approximately 3% risk of mortality and a 10% chance of relapse in HPR.^3^ Therefore, it is important for physicians to rule out more severe congenital forms of LCH and perform regular patient follow-up examinations to prevent future mortality. Considering LCH in general, among the clinical factors linked to the best prognosis, the patient presented skin-limited and monolesional disease, distal limb lesion, and absence of lesions in the mucous membrane or seborrheic area. Whether or not these specific prognostic factors apply to HPR has not been determined. Electron microscopy findings such as less than 25 percent of LCs with Birbeck granules have been considered possible clues to a favorable prognosis^3^ and the density of CD1a+ cells found in the patient’s lesion was much higher than that of CD207+ (Figs. 5 A and B). As a precautionary measure, the child will be followed up with regular physical examination until at least 2-year age, and through complete laboratory tests every 6 months and bone radiographs.^2^

It is advisable to perform the biopsy as early as possible, to obtain elements that characterize the isolated cutaneous form of this histiocytosis. In this case, it is probable that self-healing was accelerated by biopsy, as its growth and infiltration since birth, regressed soon after the first days of the procedure. Besides Hashimoto-Pritzker, there are many other cases of spontaneous tumor regression reported in the literature and several pathogenetic hypotheses for regression proposed for different types of tumors. Some of these mechanisms include apoptosis, immune system effectiveness, tumor microenvironment,^4^ and vaccine.^5^

Regulatory T-lymphocyte density does not seem to be predictive of disease evolution in LCH. However, in reports on laryngeal cancer^6^ and B-cell lymphoma,^7^ the regression observed after the biopsy was attributed to a probable activation of the immune response^6^ or a disruption in the microenvironment, eliciting immune reaction due to the trauma of the procedure.^7^

Ki67 has a role in cell division and is usually used as a prognostic marker and predictor of recurrence. The proliferative index found in the present study’s patient (62%) was high and greater than that described in the literature for HPR; so, the role of hypoxia and apoptosis should be considered. The immunohistochemical results of Ki-67 in an 11 cases study were 38.50±19.61 (mean ± SD).^8^ No differences were found in the proliferative index comparing self-regressive cutaneous LCH in 21 patients and non–self-regressive cutaneous LCH in 10 patients.^9^

The child had received BCG and Hepatitis B vaccines and their role should also be considered. It is attested the utility of BCG to induce tumor remission^5^ in bladder cancer and the DTP (Diphtheria-Tetanus-Pertussis) vaccine was described to elicit regression in metastatic melanoma.^10^

## Financial support

None declared.

## Authors’ contributions

Luciana Prates Nogueira de Lima: Study concept; data collection; writing of the manuscript; approval of the final version of the manuscript.

Carolina Viza Amorim: Data collection; approval of the final version of the manuscript.

Rachel Martins Marinho: Data collection; approval of the final version of the manuscript.

Maria Letícia Cintra: Data collection; writing of the manuscript; effective participation in the research guidance; approval of the final version of the manuscript.

Elemir Macedo de Souza: Analysis and interpretation; critical review; research guidance; approval of the final version of the manuscript.

## Conflicts of interest

None declared.
